# Real-Life Clinical Experience With Cariprazine: A Systematic Review of Case Studies

**DOI:** 10.3389/fpsyt.2022.827744

**Published:** 2022-03-17

**Authors:** Réka Csehi, Zsófia Borbála Dombi, Barbara Sebe, Mária Judit Molnár

**Affiliations:** ^1^Global Medical Division, Gedeon Richter Plc, Budapest, Hungary; ^2^Department of Psychiatry, University of Oxford, Oxford, United Kingdom; ^3^Institute of Genomic Medicine and Rare Disorders, Semmelweis University, Budapest, Hungary

**Keywords:** cariprazine, antipsychotic, case report, systematic review, psychopharmacology, partial agonist

## Abstract

**Background:**

The hierarchy of evidence coming from evidence-based medicine favors meta-analyses and randomized controlled trials over observational studies and clinical cases. Nonetheless, in the field of psychiatry, where conditions are much more complex, additional evidence coming from real-world clinical practice is necessary to complement data from these gold standards. Thus, in this systematic review, the aim is to summarize the evidence coming from clinical case reports regarding cariprazine, a third-generation antipsychotic drug that has been approved for the treatment of schizophrenia and bipolar I disorder with manic, depressive or mixed features in adults.

**Methods:**

A systematic review was performed using Embase and Pubmed databases searching for English-language cases published in peer-reviewed journals between 2000 January and 2021 September with the following search terms: (cariprazin^*^ OR “rgh-188” OR rgh188 OR vraylar OR reagila) AND (“case report^*^” OR “case report”/de OR “case stud^*^” OR “case study”/de OR “case seri^*^”).

**Results:**

After the removal of duplicates, 49 articles were retrieved via the search, from which 22 were suitable for this review. These 22 articles encompassed 38 cases from which 71% described patients with schizophrenia, 16% patients with psychotic disorders, 5% patients with mood disorder and 8% described patients with other disorders such as Wernicke-Korsakoff syndrome, borderline personality disorder and obsessive-compulsive disorder with paranoid schizophrenia. The median age of patients was 31, and half of them were female. The majority of patients (76%) started cariprazine with 1.5 mg/day, and the most common maintenance dose was 4.5 mg/day (34%) and 3.0 mg/day (29%).

**Conclusion:**

Cariprazine was found to be safe and effective in a wide range of psychiatric conditions with different symptom profiles from acute psychotic symptoms through addiction to negative and cognitive symptoms. The results are in-line with the established evidence from clinical trials, however, they also show how cariprazine can be successfully utilized for treating certain symptoms irrespective of the indication.

## Introduction

Evidence-based medicine (EBM) is a concept developed for treating patients via the integration of the best research evidence with clinical expertise, and it has gained considerable prominence in the field of psychiatry (1). According to EBM, there is a clear hierarchy of evidence based on the different research methods in which meta-analyses of randomized controlled trials (RCTs) are the highest quality evidence, while case-control and non-controlled observational studies are the least reliable sources (1). Indeed, RCTs are considered to be the gold standard in developing scientific evidence about the efficacy and safety of new interventions such as novel medications (2).

Nonetheless, the direct application of EBM to psychiatry also means to view mental disorders exactly the same way as physical disorders, which would disregard the complexity of psychiatric conditions (1). To give an example, despite the clear advantages of RCTs, one of the biggest downside concerns is generalisability, as populations involved in these trials can significantly diverge from those seen in actual clinical practices (3). Patients enrolled in clinical trials are highly selected: they go through a rigorous screening based on an extensive list of inclusion-exclusion criteria; comorbidities and concomitant medications are usually controlled; and adherence to the therapeutic product is extensively supported which does not seem feasible in real-world settings (2).

Therefore, in the field of psychiatry, it is worth considering other sources of information, such as real-world data in EBM, to complement the knowledge gained from clinical trials (1). These can be electronic health/medical records, product/disease registries, pharmacy data, electronic devices and applications, observational studies, and of particular importance to this paper, clinical case reports (4). The advantages of real-world data over clinical trials lie in that they better represent patients with a broader range of age, illness-severity, comorbidities and usage of concomitant medications, thus they help establish all the potential applications of an intervention (3). For instance, such data can provide further information regarding the effectiveness and tolerability of a drug, which is particularly important in case of new medications, where clinical experience is not yet well-established.

Thus, the focus of this review is to summarize the evidence generated by clinical case reports of cariprazine, an antipsychotic medication that has been approved for the treatment of schizophrenia in adults by the Food and Drug Administration (FDA) and the European Medicines Agency (EMA) (5), as well as for the manic/mixed and depressive episodes associated with bipolar I disorder in adult patients by the FDA (6). Furthermore, two Phase III clinical trials found positive results for the adjunctive treatment of major depressive disorder (MDD) (7). Cariprazine is a D_3_-preferring partial agonist at the dopamine D_2_/D_3_ receptors and at the serotonin 5-HT_1A_ receptors, while acting as an antagonist at the serotonin 5-HT_2B_ receptors (8). There are two major active metabolites of cariprazine, desmethyl cariprazine (DCAR) and didesmethyl cariprazine (DDCAR), both pharmacologically equipotent to cariprazine and known to be jointly responsible for the overall therapeutic effect (9, 10).

The clinical development programme of cariprazine included eight Phase II/III clinical trials with acute schizophrenia patients (11–18), four Phase II/III clinical trials including patients with bipolar I depression (19–22), three Phase II/III clinical trials (23–25) and one safety study (26) with bipolar mania patients as well as with patients with MDD (27–30). In addition, there was an observational study conducted in Latvia that explored the effectiveness and tolerability of cariprazine in schizophrenia patients with predominant negative symptoms who had inadequate response to previous antipsychotic medications (31). All in all, the findings of these trials suggest that cariprazine is a safe and effective treatment for patients with schizophrenia (1.5–6 mg/day) (32), bipolar disorder (bipolar mania: 3–6 mg/day; bipolar depression: 1.5–3 mg/day) (32) and more recently, MDD as an add-on therapy (7). Of note, cariprazine is the only antipsychotic that has proven efficacy over an active comparator in the treatment of predominant negative symptoms (18).

## Methods

### Search Strategy

This systematic review was performed in accordance with the Preferred Reporting Items for Systematic Reviews and Meta-Analyses (PRISMA) statement (33). A literature search was conducted in two databases, Embase and PubMed, looking for English language articles published between January 2000 and September 2021 with the following search terms: (cariprazin^*^ OR “rgh-188” OR rgh188 OR vraylar OR reagila) AND (“case report^*^” OR “case report”/de OR “case stud^*^” OR “case study”/de OR “case seri^*^”). Searches by hand and via the reference section of published case reports and previous review papers were further performed in order to identify relevant studies in addition to the ones identified by the database search.

### Inclusion and Exclusion Criteria

Titles and abstracts of identified articles were screened to determine eligibility for this systematic review; R.C. and Z.B.D. conducted the screening separately and then jointly agreed on which ones to include. During the screening process, the following inclusion criteria were considered: (1) case report involving one or more human subject, (2) treatment with cariprazine. In case cariprazine was only mentioned in the medical history of the patient, or insufficient data was provided regarding the extent of cariprazine treatment i.e., dosing strategy, titration scheme, or timeline, then the article was excluded.

### Data Analysis

Data retrieved from the case reports was summarized in tables and figures. Diagnoses, sex, age, starting dose and maintenance dose were analyzed using descriptive statistics. In the table summarizing the cases, a column titled “Problem” was created to describe the symptoms that led to the switch to/initiation of treatment with cariprazine. Thus, this does not necessary report on all the symptoms the patients experienced. The other table that summarizes outcomes with cariprazine reports on all the effects that were attributed to cariprazine treatment by the authors.

## Results

### Search Results

**Figure 1:**
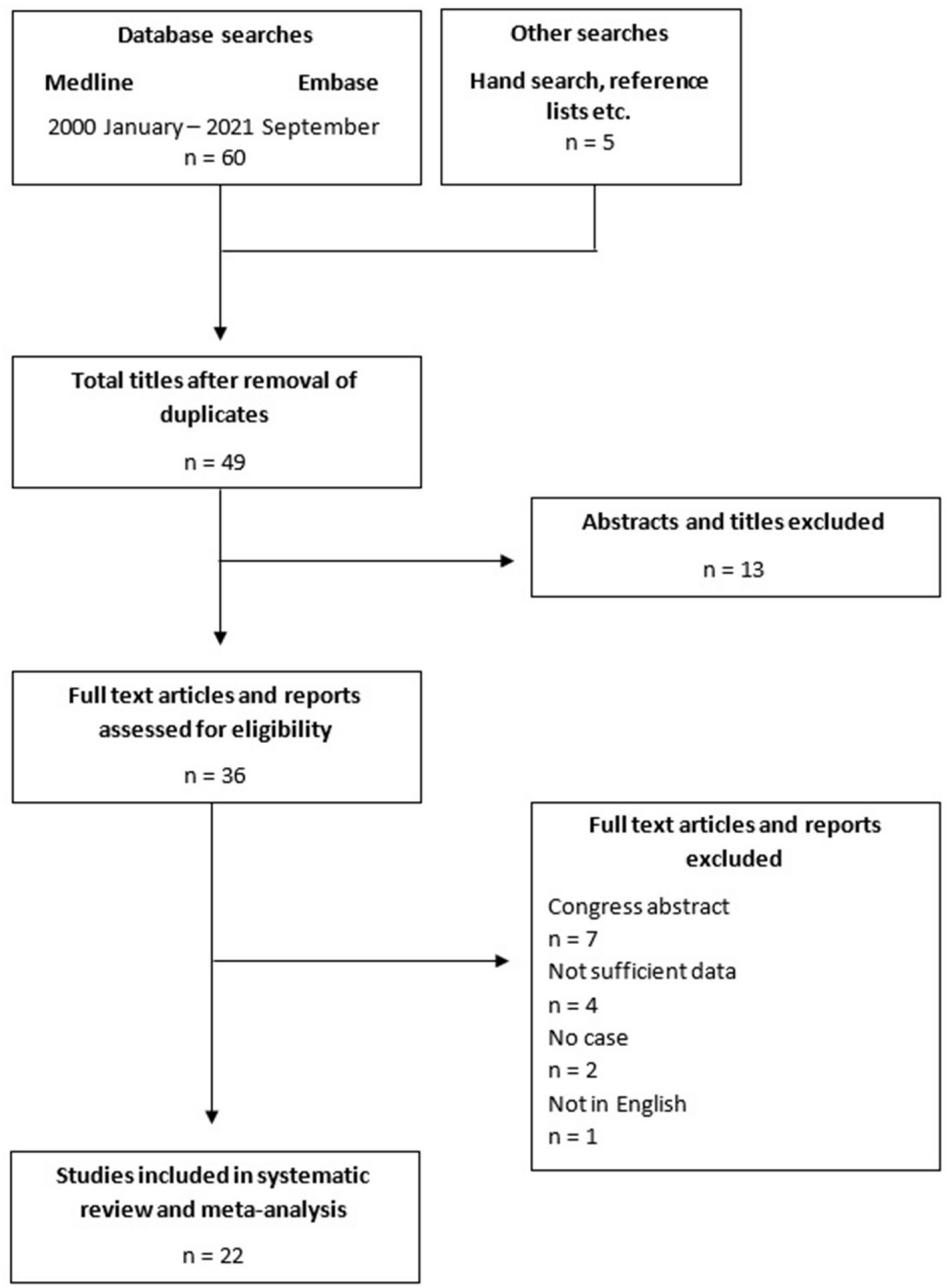
PRISMA chart.

Altogether, 60 articles were retrieved via database search, while 5 articles were found via hand search. After the removal of duplicates, 49 articles remained. Based on the titles and abstracts, 13 articles were excluded, leaving 36 articles for assessment of eligibility. After reading the full text, further 14 articles were excluded due to the following reasons: congress abstract (*n* = 7); insufficient data on cariprazine (*n* = 4); not a case report (*n* = 2); and not written in English language (*n* = 1). In the end, 22 articles were included and analyzed in this systematic review, which encompassed a total of 38 cases (Figure 1).

### Overview of Results

**Figure 2:**
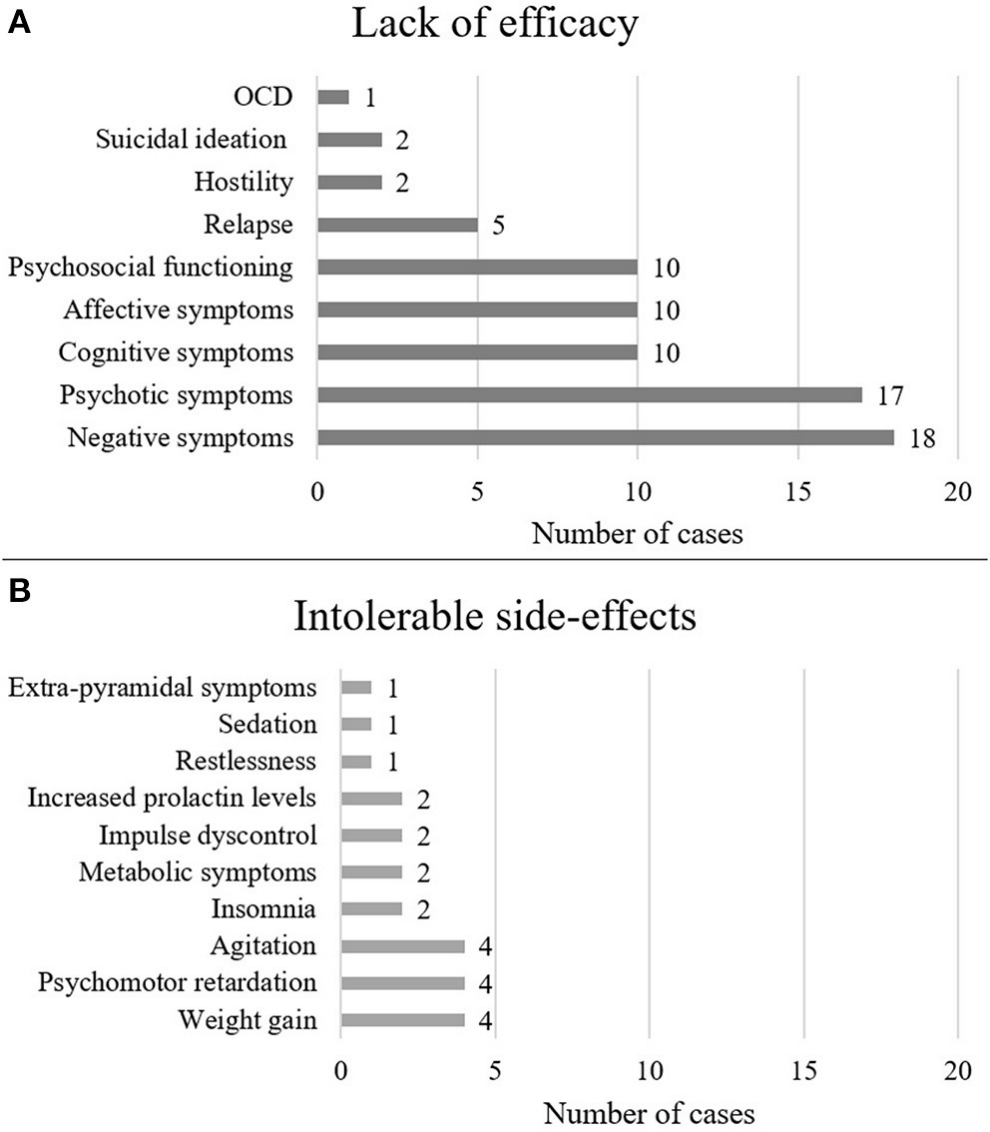
Reasons for switching to cariprazine The figure shows the number of cases in which cariprazine treatment was initiated for the given symptom **(A)** or the given side-effect **(B)**.

**Figure 3:**
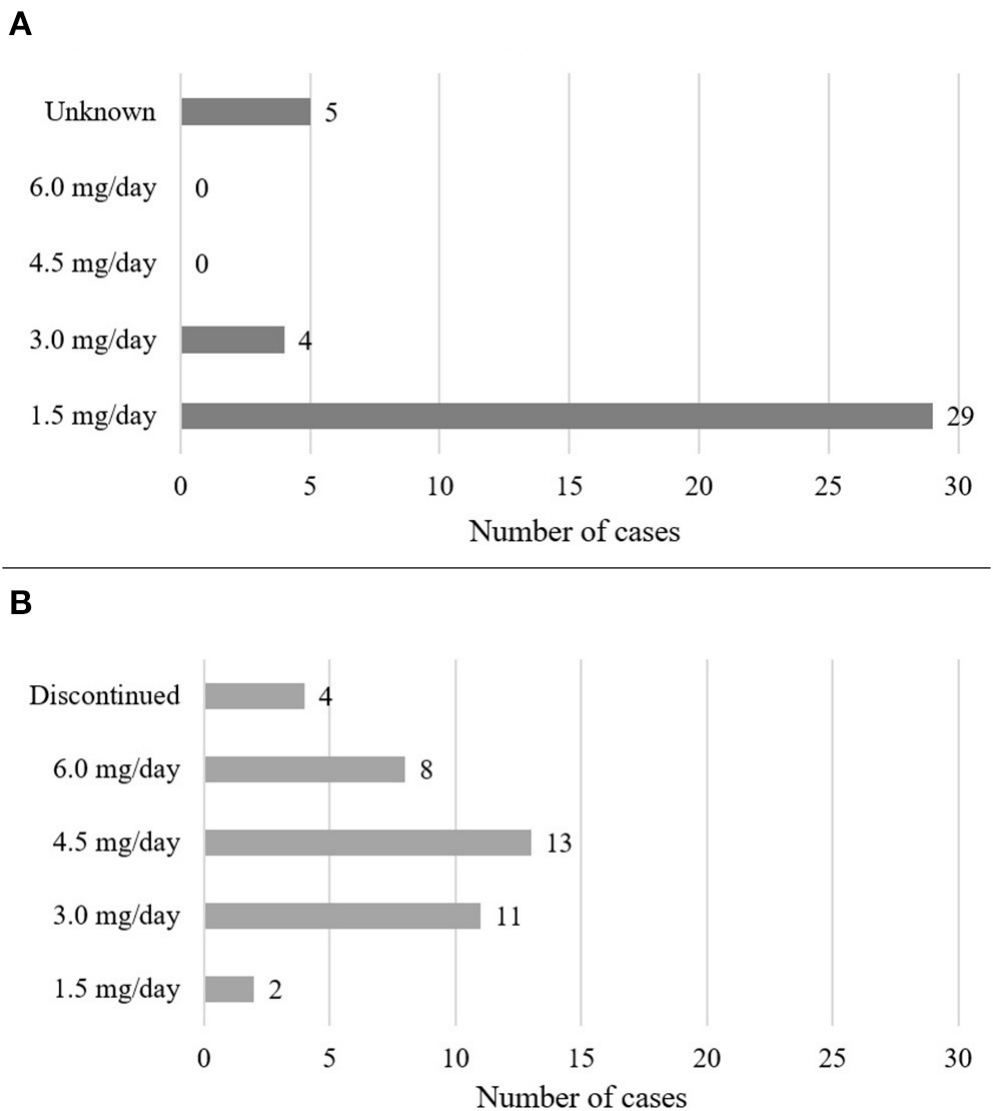
Dosing of cariprazine. The figure shows the starting **(A)** and maintenance **(B)** doses of cariprazine.

Demographics are summarized in Table 1. Out of the 38 cases, most patients were diagnosed with schizophrenia (*n* = 27, 71%), followed by psychotic disorders (*n* = 6, 16%), mood disorders (*n* = 2, 5%) and other disorders (*n* = 3, 8%) such as Wernicke-Korsakoff syndrome, borderline personality disorder and obsessive-compulsive disorder. There were 19 female and 18 male cases, and one was not specified (2.6%). The mean age of patients was 33.8 years, the median was 31 years. Dosing and clinical characteristics and are presented in Figures 2, 3. The starting dose of cariprazine was 1.5 mg/day in the majority of cases (*n* = 29, 76.3%); the rest of the patients started with 3.0 mg/day (*n* = 4, 10.5%) or it was not specified (*n* = 5, 13.5%). Most commonly, the maintenance dose was 4.5 mg/day (*n* = 13, 34.2%), followed by 3.0 mg/day (*n* = 11, 28.9%), 6.0 mg/day (*n* = 8, 21.1%) and 1.5 mg/day (*n* = 2, 5.3%). In 4 cases (10.5%), cariprazine treatment was discontinued. In most cases, patients were experiencing negative (*n* = 18, 47.4%) and psychotic (*n* = 17, 44.7%) symptoms before switching to cariprazine, followed by affective (*n* = 10, 26.3%) and cognitive (*n* = 10, 26.3%) symptoms, as well as relapse (*n* = 5, 13.2%). In terms of tolerability problems, most patients suffered from psychomotor symptoms, weight gain and agitation (all *n* = 4, 10.5%). Table 2 gives a detailed overview of each case, including sex, age, diagnosis, problem, starting and maintenance dose, titration strategy, concomitant medication, as well as provides the reference of each case. Table 3 summarizes the outcome of cariprazine treatment of each case, broken down into symptoms.

**Table 1:** Summary of results.

**Number of cases**	
Total, *n*	**38**
**Diagnosis**, ***n*** **(%)**	
**Schizophrenia**	**27 (71.1)**
Schizophrenia	13 (34.2)
Paranoid schizophrenia	8 (21.1)
Schizophrenia/schizoaffective with substance abuse	5 (13.2)
Disorganized schizophrenia	1 (2.6)
**Psychotic disorders**	**6 (15.8)**
Early psychosis	3 (7.9)
Psychosis	2 (5.3)
Acute polymorphic psychotic disorder	1 (2.6)
**Mood disorders**	**2 (5.3)**
Bipolar I disorder	1 (2.6)
Major depression	1 (2.6)
**Other**	**3 (7.9)**
Wernicke-Korsakoff syndrome	1 (2.6)
Borderline personality disorder	1 (2.6)
Obsessive-compulsive disorder with paranoid schizophrenia	1 (2.6)
**Sex**, ***n*** **(%)**	
Male	18 (47.4)
Female	19 (50.0)
Not specified	1 (2.6)
**Age**	
Mean	33.8
Median	31

*The bold values are the values of the category e.g. schizophrenia, which are under this value*.

**Table 2:** Summary of cases.

**References**	**No**.	**Sex**	**Age**	**Diagnosis**	**Problem**	**Starting dose**	**Titration strategy**	**Maintenance dose**	**Concomitant** **medication**
Amore and Aguglia (34)	Case 1	Not specified	24	Schizophrenia	Negative, cognitive, and mild psychotic symptoms with risperidone treatment, reduced psychosocial functioning	1.5 mg/day	3.0 mg/day on day 15	3.0 mg/day	Risperidone gradually discontinued
Aubel (35)	Case 2	Female	59	Paranoid schizophrenia	Negative and psychotic symptoms, psychomotor retardation, reduced psychosocial functioning	1.5 mg/day	3.0 mg/day on day 4 and 4.5 mg on day 14	4.5 mg/day	Risperidone gradually discontinued
	Case 3	Male	31	Paranoid schizophrenia	Persistent negative symptoms, psychomotor retardation	1.5 mg/day	3.0 mg/day on day 4	4.5 mg/day	Amisulpride and then 2 months later clozapine gradually discontinued
	Case 4	Male	32	Paranoid schizophrenia	Desired switch to cariprazine due to psychotic symptoms and suicidal ideation	1.5 mg/day	3.0 mg/day on day 2 and 4.5 mg/day on day 3	4.5 mg/day	Aripiprazole and risperidone gradually discontinued
Carmassi et al. (36)	Case 5	Male	39	Schizophrenia with substance abuse (alcohol, cocaine, THC, MDMA)	Negative, cognitive, and psychotic symptoms, reduced psychosocial functioning	1.5 mg/day	3.0 mg/day on day 5, 4.5 mg/day on day 9, and 6.0 mg/day on day 13	6.0 mg/day	Aripiprazole gradually discontinued, benzodiazepine
	Case 6	Male	20	Schizophrenia with substance abuse (cocaine)	Psychotic and affective symptoms, restlessness, insomnia, suicide attempt, reduced psychosocial functioning	1.5 mg/day	3.0 mg/day on day 7	4.5 mg/day	Quetiapine gradually discontinued, biperiden 4 mg/day
Rodriguez Cruz et al. (37)	Case 7	Male	30	Schizophrenia with substance abuse (amphetamine, cannabis)	Psychotic, negative, and cognitive symptoms, reduced psychosocial functioning	1.5 mg/day	6.0 mg/day on day 9	4.5 mg/day	Gradual down-titration of haloperidol over 2 weeks, quetiapine, add-on clonazepam, propranolol
De Berardis et al. (38)	Case 8	Female	29	Schizophrenia	Symptomatic despite clozapine 450 mg/day and amisulpride 800 mg/day treatment with weight gain	1.5 mg/day	3.0 mg/day after a week	3.0 mg/day	Clozapine 400 mg/day
	Case 9	Male	35	Schizophrenia	Symptomatic despite clozapine, weight gain	1.5 mg/day	3.0 mg/day after three weeks	3.0 mg/day	Clozapine 350 mg/day, then reduced to 300 mg/day
De Berardis et al. (39)	Case 10	Female	21	Early psychosis	Psychotic, negative, and cognitive symptoms, increased sedation, and appetite despite olanzapine treatment	1.5 mg/day	3.0 mg/day on day 4, 4.5 mg/day around day 30	4.5 mg/day	–
	Case 11	Male	19	Early psychosis	Psychotic, negative, cognitive, and affective symptoms, insomnia, and impulse dyscontrol	1.5 mg/day	3.0 mg/day after a few days, 4.5 mg/day and then 6.0 mg/day after 14 days	6.0 mg/day	Alprazolam 1 mg/day
De Berardis et al. (40)	Case 12	Male	26	Obsessive-compulsive disorder with paranoid schizophrenia	Persistent OCD symptoms despite paliperidone treatment	1.5 mg/day	3.0 mg/day on day 7	3.0 mg/day	Paliperidone oral suspended, add-on paliperidone long-acting injectable 100mg
Dieci et al. (41)	Case 13	Male	54	Major depression	Affective symptoms	1.5 mg/day	Not specified	1.5 mg/every second day	Citalopram 40 mg/day
Di Sciascio and Palumbo (42)	Case 14	Male	26	Schizophrenia	Psychotic relapse, and negative and affective symptoms	1.5 mg/day	3.0 mg/day on day 2	3.0 mg/day	Risperidone discontinued in 2 days
	Case 15	Female	22	Disorganized schizophrenia	Relapse due to discontinuation of previous therapy (weight gain and metabolic syndrome), cognitive and psychotic symptoms, reduced psychosocial functioning	1.5 mg/day	6.0 mg/day	6.0 mg/day	Olanzapine gradually discontinued in 2 weeks
Grant and Chamberlain (43)	Case 16	Male	42	Borderline personality disorder	Affective symptoms, hostility, and impulsivity	3.0 mg/day	4.5 mg/day after 2 weeks, 6.0 mg/day after 3 weeks	6.0 mg/day	–
Halaris and Wuest (44)	Case 17	Male	37	Schizoaffective disorder with substance abuse (alcohol and tobacco)	Metabolic syndrome with olanzapine	Not specified	3.0 mg/day and then a year later 4.5 mg/day	4.5 mg/day	Olanzapine discontinued over 2 months
Heck et al. (45)	Case 18	Female	30	Paranoid schizophrenia with substance use disorder	Relapse followed by patient's request to discontinue quetiapine	1.5 mg/day	3.0 mg on day 6	Cariprazine was reduced to 1.5 mg/day 3 days after the onset of akathisia (day 16). Another 2 days later, cariprazine was stopped.	Quetiapine 300 mg reinitiated on day 5
	Case 19	Male	22	Paranoid schizophrenia	Negative symptoms despite risperidone treatment	1.5 mg/day	3.0 mg/day after 2 weeks, then 10 days later reduced to 1.5 mg/day	1.5 mg/day	Risperidone 0.5–3 mg/day, biperiden 4 mg/day (both discontinued)
	Case 20	Male	52	Paranoid schizophrenia	Psychotic symptoms due discontinuation of medication and history of severe negative symptoms	1.5 mg/day	3.0 mg/day 1 week later, 4.5 mg/day another 5 days later	4.5 mg/day	Pipamperone 40 mg/day, then olanzapine 10 mg/day added and pipamperone discontinued
	Case 21	Female	22	Paranoid schizophrenia	Hyperprolactinemia under aripiprazole 10 mg/d and amisulpride 250 mg/d.	1.5 mg/day	Increased to 3.0, 4.5, and 6.0 mg/day after 2, 4, and 12 weeks, respectively	6.0 mg/day	–
Jimoh et al. (46)	Case 22	Female	32	Wernicke-Korsakoff syndrome	Psychotic, cognitive, and negative symptoms, psychomotor retardation despite aripiprazole treatment, reduced psychosocial functioning	Not specified	Not specified	3.0 mg/day	Not specified
Kapulsky and Brody (47)	Case 23	Male	33	Schizophrenia	Psychotic and predominantly negative symptoms despite clozapine 225 mg treatment	Not specified	Up to 6.0 mg/day in a week	Discontinued due to urinary retention	–
Mencacci et al. (48)	Case 24	Male	51	Schizophrenia	Negative symptoms despite ziprasidone, lurasidone and risperidone treatment, reduced psychosocial functioning	Not specified	Up to 4.5 mg/day	4.5 mg/day	Haloperidol and risperidone gradually discontinued
	Case 25	Female	49	Schizophrenia	Metabolic side-effects and negative symptoms despite olanzapine treatment	Not specified	Up to 4.5 mg/day until day 21	4.5 mg/day	Olanzapine gradually discontinued, and biperiden, lorazepam, antihistamine gradually reduced
Molnar et al. (49)	Case 26	Female	23	Early psychosis	Severe negative, cognitive and psychotic symptoms, agitation, reduced psychosocial functioning	1.5 mg/day	3.0 mg/day from day 4 to 12, 4.5 mg/day from day 13	3.0 mg/day	–
Montes et al. (50)	Case 27	Male	31	Schizophrenia	Psychotic symptoms	3.0 mg/day	Not specified	3.0 mg/day	–
	Case 28	Female	54	Schizophrenia	Psychotic and affective symptoms, reduced psychosocial functioning	3.0 mg/day	6.0 mg/day on day 3	6.0 mg/day	Diazepam 10 mg
	Case 29	Female	36	Schizophrenia	Psychotic symptoms, agitation, hostility despite aripiprazole treatment	3.0 mg/day	6.0 mg/day on day 3	6.0 mg/day	Quetiapine 50 mg
Müller and Moeller (51)	Case 30	Female	38	Schizophrenia	Extrapyramidal and negative symptoms	1.5 mg/day	3.0 mg/day after 4 days, 4.5 mg/day after another week	4.5 mg/day	–
	Case 31	Female	34	Psychosis	Psychotic relapse, negative and cognitive symptoms, and increased weight	1.5 mg/day	3.0 mg on day 3 for 3 weeks	4.5 mg/day	Risperidone until 4.5 mg cariprazine
Ricci et al. (52)	Case 32	Male	25	Methamphetamine-induced psychosis	Persistent psychotic, negative and affective symptoms	1.5 mg/day	3.0 mg/day on day 4, 4.5 mg/day on day 13	3.0 mg/day	Benzodiazepine
Riedesser and Gahr (53)	Case 33	Female	46	Paranoid schizophrenia	Psychotic, affective, and psychomotor symptoms and agitation	1.5 mg/day	1.5 mg/day	Discontinued after 5 days	Clozapine 12.5 mg/day, escitalopram 10 mg/day
	Case 34	Female	62	Paranoid schizophrenia	Haloperidol, then amisulpride without sufficient antipsychotic effect	1.5 mg/day	Up to 4.5 mg/day	3.0 mg/day	Amisulpride, biperiden (later phased out), hydro-chlorothiazide, amlodipine and ramipril
	Case 35	Female	19	Acute polymorphic psychotic disorder	Hyperprolactinaemia attributed to risperidone and olanzapine	1.5 mg/day	3.0 mg/day	Discontinued after 2 weeks	Olanzapine 5 mg/day discontinued after 4 days; pantoprazole initiated
Sanders and Miller (54)	Case 36	Female	20	Bipolar I disorder, ADHD, substance use disorder (cannabis and alcohol)	Affective and cognitive symptoms and agitation	1.5 mg/day	3.0 mg/day after 3 weeks	3.0 mg/day	Quetiapine 25 mg/day, clonazepam 2 × 0.5 mg/day, methylphenidate XR 72 mg/day
Vita et al. (55)	Case 37	Female	31	Schizophrenia	Negative symptoms despite risperidone treatment	1.5 mg/day	3.0 mg/day on day 4, 4.5 mg/day on day 7	4.5 mg/day	Risperidone dose decreased by 3 mg every 3 days until full discontinuation
	Case 38	Female	27	Schizophrenia	Psychotic relapse 2 weeks after the administration of paliperidone palmitate 1-monthly long-acting therapy	1.5 mg/day	3.0 mg/day on day 4, 4.5 mg/day on day 7, 6.0 mg/day on day 10	6.0 mg/day	Paliperidone discontinued

**Table 3:** Clinical outcomes with cariprazine treatment.

**References**	**No**.	**Outcome**																		
		**Efficacy**									**Safety**									
		**Positive**	**Negative**	**Cognitive**	**Affective**	**Hostility**	**Substance abuse**	**OCD**	**Impulsivity**	**Psychosocial functioning**	**Psychomotor**	**Insomnia**	**Sedation**	**Weight gain**	**Metabolic syndrome**	**Increased prolactin levels**	**Agitation**	**EPS**	**Sexual dysfunction**	**Restlessness**
Amore and Aguglia (34)	Case 1	↓	↓	↓	↓					↑						↓		↓	↓	
Aubel (35)	Case 2	X	↓							↑	X									
	Case 3	X	X		X					↑										
	Case 4	X		↓						↑										
Carmassi et al. (36)	Case 5	↓	↓	↓			X			↑	X		↓							
	Case 6		↓	↓	↓		X			↑								+		
Cruz et al. (37)	Case 7	↓	↓				X			↑								+		
De Berardis et al. (38)	Case 8	↓	↓							↑				↓						
	Case 9	↓	↓	↓						↑				↓						
De Berardis et al. (39)	Case 10	↓	↓	↓									↓							
	Case 11	↓	↓	↓	↓				X			↓								
De Berardis et al. (40)	Case 12							X												
Dieci et al. (41)	Case 13				↓													+	X	
Di Sciascio and Palumbo (42)	Case 14	↓	↓		↓					↑										
	Case 15	X	X	X	↓		X			↑			↓	↓						
Grant and Chamberlain (43)	Case 16				↓	X			X											
Halaris and Wuest (44)	Case 17	X	↓							↑				↓	↓					
Heck et al. (45)	Case 18^*^	↓																+		+
	Case 19																	+		
	Case 20	↓	↓									↓								+
	Case 21															↑				
Jimoh et al. (46)	Case 22	↓	↓	↓						↑										
Kapulsky and Brody (47)	Case 23^*^																			
Mencacci et al. (48)	Case 24		↓	↓						↑										
	Case 25	↓		↓										↓		↓				
Molnár et al. (49)	Case 26	↓	↓	X						↑							X	+		X
Montes et al. (50)	Case 27	↓			↓															
	Case 28	↓			↓	↓				↑										
	Case 29	↓			↓	↓														
Müller and Moeller (51)	Case 30		↓															X		
	Case 31	↓	↓	↓										↓						
Ricci et al. (52)	Case 32	↓	↓				X					+								
Riedesser and Gahr (53)	Case 33^*^																+	+		
	Case 34	↓																+		
	Case 35															X		+		
Sanders and Miller (54)	Case 36			X			X			↑							X			X
Vita et al. (55)	Case 37	X	↓							↑										
	Case 38	X																		

***↑**, Increase; **↓**, Decrease; X, Absent; +, Present*.

* *Discontinued due to akathisia (case 18, 33, 35) or urinary retention (case 18)*.

### Schizophrenia

Out of the 38 cases, 27 had a diagnosis of schizophrenia (71.1%), of whom eight cases had a diagnosis of paranoid schizophrenia (29.6%), five had schizophrenia/schizoaffective disorder with substance abuse (18.5%), one patient had disorganized schizophrenia (3.7%), and in 13 cases, the subtype of schizophrenia was not specified (48.1%).

#### Initiation of Cariprazine Treatment

Cariprazine treatment was primarily initiated due to the lack of efficacy of previous medications or drug-induced side-effects. The presence of positive symptoms (*n* = 19) was one of the main reasons for switching to cariprazine. These symptoms usually emerged due to medication non-adherence and therefore relapse. Following positive symptoms, the presence of negative symptoms was the most common reason for initiating cariprazine treatment (*n* = 12), which emerged due to the lack of efficacy or as a result of previous antipsychotic treatments. Patients reported experiencing reductions in motivation, social interactions, and everyday activities. Furthermore, cognitive (*n* = 4) and affective (*n* = 4) symptoms as well as substance abuse (*n* = 4; mainly alcohol and cocaine) indicated a switch to cariprazine treatment.

Many patients experienced intolerable side effects induced by other antipsychotics, therefore cariprazine was initiated due to its favorable safety profile. Three patients experienced psychomotor retardation (*n* = 3), and in case of one patient (*case 2*) (35), it got so severe that she became fully bedridden. Furthermore, weight gain (*n* = 3) and the development of metabolic syndrome (*n* = 3) were problematic for patients; one of them (*case 15*) (42) gained 30 kg after the first year of taking olanzapine, causing increases in triglyceride and cholesterol levels. As a result, she developed social inhibition and stopped practicing her hobbies, thus impacting on her everyday life. Insomnia (*n* = 1), agitation (*n* = 2), restlessness (*n* = 1), and heightened prolactin levels (*n* = 1) were further issues for which cariprazine treatment was initiated.

The starting dose of cariprazine was 1.5 mg/day in most cases (*n* = 20, 74.1%), followed by 3.0 mg/day (*n* = 3, 11.1%), while it was not specified in four cases (14.8%). In most cases, the maintenance dose was 4.5 mg/day (*n* = 11, 40.8%), followed by 6.0 mg/day (*n* = 6, 22.2%), 3.0 mg/day (*n* = 6, 22.2%) and 1.5 mg/day (*n* = 1, 3.7%), while cariprazine was discontinued in three cases (11.1%).

#### Outcome of Cariprazine Treatment

Overall, cariprazine proved to be an effective treatment for many symptom domains: it reduced positive (*n* = 20), negative (*n* = 15), cognitive (*n* = 8) and affective symptoms (*n* = 8); reduced hostility (*n* = 2); yielded substance-abstinence (*n* = 4); and improved psychosocial functioning (*n* = 14).

Importantly, there were five patients (*cases 5, 6, 7, 17, 18*) (36, 37, 44, 45) with comorbid substance use disorder, who had several antipsychotic failures previously either due to ineffectiveness in reducing symptoms of schizophrenia and addiction, or due to severe side-effects. The patients had severely impaired psychosocial functioning and quality of life. Cariprazine yielded significant improvements in positive, negative, cognitive, and affective symptoms, it contributed to substance abstinence and had positive effects on sleep regulation, psychomotor symptoms, weight decrease and metabolic symptoms. Although cariprazine induced extrapyramidal symptoms in two cases (*case 6 and 7*) (36, 37), they were well managed by the reduction of dose (from 6 to 4.5 m g/day in *case 7*) (37) and by using concomitant medication (*cases 6 and 7*) (36, 37).

In case of four patients (*cases 14, 15, 18, 38*) (42, 45, 55), cariprazine treatment was initiated due to psychotic relapse. Three patients decided on their own to discontinue previous medication (*cases 14, 15, 18)* (42, 45): two of them (*cases 14, 15*) (42) due to undesired side-effects, such as sexual difficulties, weight gain, sedation, the emergence of negative and affective symptoms, as well as a reduction in psychosocial functioning. One patient (*case 38*) (55) switched to cariprazine due to the inefficacy of the long-acting therapy. In all cases, cariprazine effectively reduced and reversed the side-effects of previous medications without causing any other side-effect. In addition, it proved to be an efficacious medication yielding remission of psychotic, negative, affective, and cognitive symptoms.

Regarding the effects of cariprazine on psychosocial functioning, eight patients (*cases 1, 2, 5, 6, 7, 15, 24, 28)* experienced difficulties with it before cariprazine treatment. Importantly, after the initiation of cariprazine, fifteen patients (*cases 1–9, 14, 15, 17, 24, 28, 37*) experienced a significant improvement in psychosocial functioning and quality of life as a result, including re-entering school or securing a job, and being more involved in family and social activities and events.

As a result of cariprazine treatment, psychomotor retardation resolved in two cases, including the previously described patient (*case 2*) (35) who recovered from being fully bedridden, and at the time of the report, she took care of her household independently. Cariprazine also contributed to the resolution of metabolic syndrome (*n* = 1) and weight reduction of five patients, including the patient who gained 30 kg in a year due to the previous medication (*case 15*) (42). Furthermore, cariprazine demonstrated beneficial effects toward normalizing prolactin levels (*n* = 2, while increased it in one patient), sexual function (*n* = 1), and sleep patterns (insomnia *n* = 1; sedation *n* = 2). Nonetheless, it is also worth noting that cariprazine contributed to the development of agitation in one, and restlessness in two patients.

#### Side Effects and Discontinuation

Although cariprazine reduced extrapyramidal symptoms in one patient and led to its complete resolution in another, the most common side-effects induced by cariprazine were extrapyramidal symptoms (EPS), mainly akathisia, reported in six patients.

Cariprazine was discontinued in three cases (11.1%): due to akathisia in two cases (*cases 18 and 33*) and due to urinary retention in one patient (*case 23*) (47). In the latter case, cariprazine was up-titrated to 6.0 mg/day in a week when the patient complained of dysuria. The bladder scan revealed a postvoid residual urine volume of 900 mL (reference: <50–100 mL), therefore cariprazine was discontinued given the proximity of its introduction and the onset of urinary retention. After 3 days, the postvoid residual urine volume decreased to normal range. In the other patient (*case 18*) (45), 6 week after the dose increase of cariprazine from 1.5 to 3.0 mg/day, the patient complained of restlessness of lower extremities, anxiety, and an uncontrollable urge to move around. Despite a significant reduction in psychotic symptoms, cariprazine was reduced to 1.5 mg/day 3 days after the onset of akathisia, and another 2 days later, cariprazine treatment was stopped. The patient started to appear calmer and less distressed 5 weeks after the discontinuation of cariprazine, however, slight akathisia symptoms remained for another 3 weeks until her discharge. Finally, in case 33, cariprazine-treatment was initiated at 1.5 mg/day, but it was discontinued 5 days later due to cariprazine-induced akathisia, agitation, and parkinsonism of extremities. The following day, the symptoms subsided completely.

### Psychotic Disorders

Six cases discussed patients with different psychotic disorders such as early psychosis or acute polymorphic psychotic disorder. The three patients with early psychosis, two females (*case 10 and 26*) and one male (*case 11*), were between 19 and 23 years old, who all suffered from psychotic, negative, and cognitive symptoms (39, 49). In the female patients, self-neglect and social withdrawal were prominent, while the male patient showed signs of impulse dyscontrol (39, 49). All patients started their cariprazine treatment on 1.5 mg/day, which was up-titrated to the necessary dosage of 3.0, 4.5, or 6.0 mg/day (39, 49). Improvement in overall symptoms with maintained effect with cariprazine was reported in all three cases (39, 49). Remarkably, cariprazine was highly effective in eliminating impulse dyscontrol as well as treating severe and predominant negative symptoms in a drug-naïve patient (39, 49). The patients reported that “it is a long time since my thoughts were so clear” and “I feel more alive” (39). Interestingly, one of them was followed-up for 52 weeks, and was reported to be symptom-free with cariprazine (49).

The other three psychotic disorder cases included a patient with methamphetamine-induced psychosis (*case 32*) (52), psychosis (*case 31*) (51), and acute polymorphic psychotic disorder (*case 35*) (53). The latter suffered from hyperprolactinaemia due to treatment with olanzapine and risperidone and was prescribed 1.5 and then 3.0 mg/day cariprazine (53). Although serum prolactin levels normalized, due to the development of severe akathisia, cariprazine was discontinued after 2 weeks (53). The other two patients were experiencing psychotic, negative, cognitive and affective symptoms and received 1.5 mg cariprazine per day (51, 52). Cariprazine dosage was increased up to 3.0 mg/day in the patient with substance-induced psychosis with adjunctive benzodiazepines for insomnia, which resulted in an improvement in both negative and psychotic symptoms (52). Most importantly, the patient remained abstinent from methamphetamine (52). Similar improvement of symptoms on 4.5 mg/day was seen in the other patient who switched from risperidone to cariprazine and who also reported significant weight loss (16 kg) (51).

### Mood Disorders

Altogether, two cases were found that reported on patients with mood disorders. One of these cases (*case 36*) described a young patient with a diagnosis of bipolar I disorder, ADHD and substance abuse disorder (alcohol and cannabis) (54). According to the report, the patient exhibited affective and cognitive symptoms as well as agitation (54). Cariprazine 1.5 mg/day was prescribed for 3 weeks, then up-titrated to 3.0 mg/day, as no improvement was detected with the lower dose. Concomitant medications, quetiapine, clonazepam and methylphenidate XR were also taken by the patient (54). After three additional weeks, the patient improved a lot and more importantly, she remained substance free even after 27 months (54). The other case (*case 13*) reported on a man with MDD who had been taking several different antidepressant medications without significant response (41). Cariprazine 1.5 mg/day was initiated as add-on therapy to his current regimen and led to partial improvements of depressive symptoms and significant improvement of sexual functioning after 30 days (41). Due to mild akathisia, cariprazine dose was reduced to 1.5 mg per every second day, to which akathisia disappeared (41).

### Other Disorders

Finally, three cases outside the approved and clinically studied indications were determined. The first (*case 12*) described a patient with obsessive-compulsive disorder (OCD), a history of paranoid schizophrenia and current treatment of long-acting paliperidone and oral paliperidone 6 mg (40). After suspending oral paliperidone, introducing cariprazine 1.5 mg/day, and then up-titrating it to 3.0 mg/day within a week, the patient's symptoms decreased without any adverse effects (40). The second patient (*case 16*) was diagnosed with borderline personality disorder and reported to suffer from affective symptoms, hostility, and impulsivity (43). Cariprazine dose was continuously increased from 3.0 to 4.5 mg/day, and then to 6.0 mg/day after the improvement of affective symptoms (43). Seven months later, no signs of impulsivity, hostility or adverse events were detected. (43). The third patient (*case 22*) had Wernicke-Korsakoff syndrome with psychotic, cognitive, and negative symptoms as well as psychomotor retardation despite aripiprazole treatment (46). After switching to cariprazine 3.0 mg/day, improvements in both psychotic and negative symptoms were detected without any side-effects, which was followed by cognitive improvement 3 months later (46).

## Discussion

This is the first systematic review that summarizes the real-life effectiveness and tolerability of cariprazine based on published case studies. The results of the review indicate that cariprazine is an effective treatment option for a wide range of symptoms in several psychiatric conditions including schizophrenia, psychotic disorders, mood disorders and even borderline personality disorder. In addition, it has been also shown that cariprazine is well-tolerated by the majority of patients and it has the potential to reverse some of the side effects such as weight gain or hyperprolactinemia that are typically caused by other antipsychotic medications. The relevance of these results in relation to the wider literature is discussed below.

### Treatment Initiation and Dosing

In almost all cases, cariprazine was initiated at 1.5 mg/day dose and then up-titrated to the necessary dosage, most commonly to 4.5 mg/day. This dosing strategy is recommended by the summary of product characteristics (SmPC) (5) and by other expert opinion panels as well (56). Recently, an article by Rancans and colleagues summarized the evidence regarding cariprazine dosing and came to similar conclusions (57).

### Effectiveness in a Wide Range of Symptoms

#### Psychotic and Manic Symptoms

Irrespective of the diagnosis, cariprazine was found to reduce or even resolve psychotic symptoms, such as hallucinations, delusions, or disruptive behavior, in many different cases from early psychosis to psychotic relapses. Although most dopamine D_2_/D_3_ partial agonists are perceived as being “weak” in terms of addressing positive symptoms compared to D_2_ antagonists, such examples show that with adequate doses, a partial agonist can also initiate and maintain the necessary therapeutic effect. Indeed, the pooled results of the three short-term Phase II/III clinical trials reported cariprazine to be significantly better than placebo in reducing positive symptoms in acute patients as measured by mean change form baseline to week 6 in the Positive and Negative Syndrome Scale (PANSS)-derived Marder Positive Symptom factor score (58). Short-term trials demonstrated the efficacy of cariprazine in the treatment of bipolar mania or mixed episodes. Change from baseline to week 3 in the Young Mania Rating Scale (YMRS) scores was significantly greater in the cariprazine group than in the placebo group (23–25). What is more, the efficacy of cariprazine was extended over the longer-term, as shown by a 16-week-long study (26). *Post-hoc* analysis of these studies showed significant improvements on the Young Mania Rating Scale (YMRS) scores (manic symptoms) and numerically greater improvements in the MADRS scores (depressive symptoms) at week 3 in the cariprazine-group, compared to the placebo group (59). This effect is attributed to the high affinity of cariprazine for D_2_ receptors (60, 61), as overactive dopamine release at the D_2_ postsynaptic receptors is hypothesized to induce the positive symptoms of schizophrenia (62, 63) as well as the manic symptoms of bipolar I disorder (64).

#### Hostility

Similarly to psychotic symptoms, hostility was also reported in a few of the reviewed acute cases (43, 50). In these patients, anger outbursts and other hostile behavior reduced significantly after the initiation of cariprazine treatment (43, 50). Again, the pooled results of three short-term clinical trials with acute schizophrenia patients also showed that cariprazine, compared with placebo, produced significantly greater improvement in hostility in patients with acute exacerbation of schizophrenia, with greatest effect in patients with the highest level of baseline hostility (65). Importantly, based on the results of this review, patients who stayed on cariprazine for a longer period (up to 27 months) did not experience relapse, which is in line with the results of the relapse-prevention study of cariprazine (17). The trial showed that time to relapse was significantly longer in the cariprazine- compared to placebo-treated patients and occurred in <25% of cariprazine- and more than 45% of placebo-treated patients (17). Thus, it can be stated that cariprazine might be a good choice of medication for acute as well as maintenance treatment of several psychiatric conditions.

#### Negative Symptoms

Among other antipsychotics, cariprazine has the highest affinity to D_3_ receptors, even greater than that of dopamine itself (8). Given other antipsychotics' low affinity to D_3_, in the presence of dopamine, cariprazine is the only antipsychotic that is able to block the D_3_ receptors in the living brain (66). D_3_ actions translate into efficacy against negative and cognitive symptoms, improving mood and regulating motivation and reward-related behavior (67). Thus, it is not surprising that cariprazine is currently the only antipsychotic medication that was found to be significantly better in the treatment of predominant negative symptoms than an active comparator, risperidone (18). Indeed, in many cases, cariprazine was prescribed due to residual negative symptoms which were resolved in all cases. The effectiveness of cariprazine in real life was also assessed in an observational study that was conducted in Latvia involving 116 patients with predominant negative symptoms (31). The results also supported the notion that cariprazine is a valid treatment option for those patients who have residual negative symptoms with ongoing antipsychotic treatment (31). Nonetheless, this effect is not exclusive to patients with predominant negative symptoms; in the pooled *post-hoc* analysis of three short-term Phase II/III clinical trials, cariprazine was found to be also significantly better than placebo in reducing negative symptoms in acute patients as measured by the mean change from baseline to week 6 in the PANSS Marder Negative Symptom factor score (58).

#### Cognitive Symptoms

Neurocognitive deficits are also a core feature of many neuropsychiatric disorders, including schizophrenia and bipolar disorder, and are associated with reduced psychosocial functioning and worse illness prognosis (68, 69). Evidence suggest that cognitive effects are further mediated by the D_3_ receptors (67). Indeed, in many of the presented cases, cariprazine effectively enhanced patients' cognition, further contributing to improved quality of life. In support of its potential advantage for treating cognitive symptoms, cariprazine yielded significantly greater improvements in cognition than an active comparator, risperidone, as measured by both the PANSS Meltzer Cognitive subscale and the PANSS-derived Marder factor for Disorganized Thoughts in a 26-week-long Phase IIIb clinical trial (70). However, cariprazine did not only improve cognitive symptoms in the long-term, but also in the short-term studies; pooled data from three 6-week, phase II/III trials demonstrated the superiority of cariprazine over placebo in the reduction of cognitive symptoms, as measured by the PANSS-derived Marder Disorganized Thought factor—this effect was driven by all 7 factor items (58). In bipolar disorder, pooled analysis of three Phase II/III trials showed that 3 weeks of cariprazine treatment significantly enhanced cognition compared to placebo in patients with manic/mixed episodes, as measured by the PANSS Cognitive subscale (71). Finally, pooled analysis of three Phase II/III trials demonstrated similar improvements in cognition for patients with bipolar I depression at week 6 of cariprazine treatment, as measured by the change in Concentration item score of the Montgomery-Åsberg Depression Rating Scale (MADRS) (72).

#### Addiction

The observed effect of cariprazine in the reduction of substance use can also be attributed to the role of D_3_ receptors (54). D_3_ receptors are highly expressed in limbic areas forming the “reward circuitry”, implying their involvement in the mediation of motivation and emotions—all heavily involved in the pathophysiology of addiction (73). To date, in addition to the presented cases here, evidence for the effectiveness of cariprazine in substance abuse comes from animal studies: in rats, cariprazine reduced the rewarding effects of cocaine and prevented relapse, and its effect was 20 times more potent than that of another partial agonist, aripiprazole (74). Two clinicals trials have been initiated to uncover the effectiveness of cariprazine in substance use disorder, although the results are not yet available. A Phase II, placebo-controlled study is focusing on how cariprazine (1.5 vs. 3.0 mg/day) affects the brain and behavior in cocaine use disorder, using fMRI (75). Furthermore, a Phase IIa, randomized, placebo-controlled pilot study will shed light on how low-dose cariprazine (1.5 mg/day) treatment impacts on cocaine use in patients with comorbid opioid use disorder who are medically stable and have already been on a stable dose of buprenorphine/naloxone treatment (76). Currently, a narrative review has investigated the potential of cariprazine in the treatment of substance use disorder in patients with bipolar disorder and concluded that based on the evidence and the receptor profile of cariprazine, it is a potential pharmacological treatment option for this patient population (77). However, further studies are warranted to validate this rationale-based postulation.

#### Affective Symptoms

Although only one case report described the efficacy of cariprazine in bipolar disorder and another in MDD, the positive outcomes of these cases and the potential of cariprazine for the treatment of affective symptoms are further supported by the findings of clinical trials. A *post-hoc* analysis of three pivotal studies in bipolar depression was conducted to evaluate the efficacy of cariprazine in patients with or without manic features (78). For patients with manic symptoms, both 1.5 and 3.0 mg/day cariprazine yielded significant improvements on the MADRS scores, while for patients without manic symptoms, the 1.5 mg/day cariprazine dose was significantly more effective than placebo (78). Furthermore, two Phase III clinical trials found positive results for the efficacy of cariprazine in MDD as adjunctive treatment (7): compared to placebo, patients treated with 1.5 mg/day cariprazine showed significantly greater change from baseline to week 6 in the MADRS total scores. Dopamine D_3_ and serotonin 5-HT_1A_ receptors have been implicated in mood disorders; given the high affinity of cariprazine to these receptors, its antidepressant efficacy may be mediated by these receptors (67, 79).

#### Psychosocial Functioning

Psychosocial functioning remains one of biggest causes of disability in patients with serious mental illness (80). Patients have severe social and occupational dysfunctions, difficulty attending to everyday tasks due to clinical symptoms (especially negative, affective and cognitive) or comorbid conditions, which have detrimental effects on their quality of life—yet, it remains a large unmet need (81). Furthermore, its evaluation is not standardized, as there is no consensus definition of psychosocial functioning (82). This was reflected in the presented cases as well: 10 patients reported reduced psychosocial functioning for which cariprazine treatment was initiated. In clinical trials of schizophrenia patients with predominant negative symptoms, cariprazine significantly improved patient functionality from week 10 onwards compared to risperidone, as measured by the Personal and Social Performance scores, driven by significant changes in all three relevant subdomains (18). In patients with bipolar I depression, despite depressive symptoms being one of the main contributors to reduced functioning, the resolution of depressive symptoms is not enough; impaired psychosocial functioning persists during periods of euthymia (83). Studies of patients with bipolar I depression showed that cariprazine significantly improved psychosocial functioning, as indicated by 5 of 6 subscale scores of the Functioning Assessment Short Test (Autonomy, Occupational Functioning, Cognitive Functioning, Leisure Time, and Interpersonal Relationships). Therefore, cariprazine is a good pharmacological treatment choice, as it does not only improve clinical symptoms, but also contributes to improved patient functioning, which is often more important for patients than the resolution of symptoms. These findings from the clinical trials are in line with the reported cases here, as improved psychosocial functioning and quality of life was reported in 20 patients.

### Cariprazine Is Well-Tolerated Compared to Other Medications

Cariprazine proved to be a safe and tolerable medication based on the results of this review and previous trials as well. Its activity at serotonin 5-HT_2B_ receptors (antagonist), 5-HT_1A_ receptors (partial agonist), and activity with lower affinity for 5-HT_2A_, 5-HT_2C_, histamine H_1_, and α_1_ receptors may have implications for the gentle safety profile of cariprazine on metabolic, sedative and hyperprolactinaemia-related side-effects (84).

#### Metabolic Symptoms and Weight

Second-generation antipsychotics have been associated with the development of metabolic symptoms and increase in weight (85). Weight gain is among the most concerning side-effects for patients, and metabolic syndrome contributes to a reduction in quality of life and satisfaction with care, and contributes to the premature mortality of patients with serious mental illness, compared to the general population (80). Cariprazine led to a reduction in weight in six patients, sometimes even without dieting or exercising, who all gained weight due to their previous antipsychotic medication. Although only one case reported an improvement in metabolic symptoms (other than weight loss), safety studies confirm that cariprazine is metabolically neutral in the approved dose-range, and is comparable to placebo (84). Specifically, cariprazine caused only slight changes in weight, hyperlipidaemia, hyperglycaemia and diabetes mellitus and no dose-response relationship was observed in the long-term studies (86).

#### Extrapyramidal Symptoms

Akathisia is another common side-effect of antipsychotics, and it is associated with increased risk of suicide or aggressive behavior, treatment discontinuation, and ultimately, lower quality of life (87). In the presented cases, despite reducing EPS symptoms in 2 patients (*cases 1 and 30*) (34, 51), these were the most commonly reported side-effects of cariprazine treatment (*n* = 9). The symptoms were managed either by dose-reduction or the administration anti-akathisia medication. These EPS-management strategies were also applied in clinical trials of patients with schizophrenia, as well as they are recommended by experts (84, 88). Cariprazine was discontinued due to EPS (akathisia) in three cases (*case 18, 33, 35*) (45, 53): 5 days (*cases 18 and 33*) (45, 53) and 2 weeks (*case 35*) (53) after the onset of symptoms. In the discontinuation cases, only one case (*case 18*) (45) adapted one of the above-mentioned strategies (dose reduction), although after 2 days only on the lower dose, cariprazine was stopped. In case of the emergence of akathisia in cariprazine trials, the median time to resolution of akathisia was 17 days when anti-akathisia medication was added, with 85% of events resolving. The median time to resolution in case of down-titration of cariprazine was 15 days with over 90% of event resolving. Therefore, it is recommended to first try one of the above-mentioned strategies and give it some time before deciding to completely withdraw cariprazine. In order to minimize the risk of developing akathisia, it is also recommended to adapt a slower up-titration strategy when introducing cariprazine, as well as stick with lower doses if possible, as higher doses are associated with greater risk of developing akathisia (18, 84).

#### Prolactin Level Changes

Five patients experienced increased prolactin levels or hyperprolactinaemia as well as sexual side effects in response to previous antipsychotic treatment (olanzapine and amisulpride) which was reduced after cariprazine was prescribed and taken by the patients (34, 41, 45, 48, 53). This is not surprising given the fact that no treatment-emergent adverse events of prolactin elevation and low rate of sexual dysfunction were found in the pooled analysis of eight schizophrenia studies (84). However, in *case 21* (45), cariprazine was administered to a female patients who experienced high prolactin elevation due to aripiprazole and amisulpride. Since she had a family history of breast cancer, she was given cariprazine as a precautionary measure. Although prolactin levels decreased, 13 months after the start of cariprazine treatment, prolactin levels elevated, however, it was classified as non-serious and cariprazine treatment was continued under regular endocrinological and gynecological surveillance.

#### Sleep Disturbances

Next to akathisia, insomnia was the second most frequently occurring side-effect of cariprazine with a dose-response effect observed in the pooled safety studies (84). Since cariprazine is rather an activating substance, it is not surprising that it improved sedation in three of the reviewed cases.

#### Agitation

Agitation and restlessness were also common symptoms in patients before cariprazine treatment, however, a reduction was experienced in most of them in response to cariprazine (49, 54). Yet, in three cases, agitation was induced by cariprazine, which resulted in withdrawing cariprazine treatment (45, 53). Throughout the clinical development program of cariprazine, restlessness was described as an adverse event that can as a result of cariprazine treatment in a dose-response manner (84).

### Limitations

The biggest limitation of the present systematic review is publication bias. It is well-known that successful case reports are much more likely to be submitted by authors than unsuccessful ones, except those cases where the patient develops a serious side effect (89). Indeed, in the present review, only four articles (10.5%) reported cariprazine to be unsuccessful compared to 34 cases (89.5%) where cariprazine was effective and well-tolerated. Nonetheless, the aim of this systematic review is not to determine the efficacy and safety of cariprazine, but to provide additional information on its use in clinical practice.

## Conclusions

Cariprazine was found to be safe and effective in a wide range of psychiatric conditions with different symptom profiles from acute psychotic symptoms through addiction to negative and cognitive symptoms. The results are in-line with the established evidence from clinical trials, however, it also shows how cariprazine can be successfully utilized for the treatment of many symptoms, irrespective of the indication. Although according to evidence-based psychiatry, case reports are lower quality evidence, this systematic review shows that they can contribute to the overall scientific knowledge and support what has been established in clinical trials.

## Data Availability Statement

The original contributions presented in the study are included in the article/supplementary material, further inquiries can be directed to the corresponding author/s.

## Author Contributions

RC, ZBD, BS, and MM contributed to conception of the manuscript. RC and ZBD wrote the first draft of the manuscript. All authors contributed to manuscript revision, read, and approved the submitted version.

## Funding

Gedeon Richter provided funds for the open access publication fees.

## Conflict of Interest

The authors declare that this study received funding from Gedeon Richter Plc. The funder had the following involvement in the study: cover of the open access fees. RC, ZBD, and BS are employees of Gedeon Richter Plc. The remaining author declares that the research was conducted in the absence of any commercial or financial relationships that could be construed as a potential conflict of interest.

## Publisher's Note

All claims expressed in this article are solely those of the authors and do not necessarily represent those of their affiliated organizations, or those of the publisher, the editors and the reviewers. Any product that may be evaluated in this article, or claim that may be made by its manufacturer, is not guaranteed or endorsed by the publisher.
